# Male Courtship Rate Plasticity in the Butterfly *Bicyclus anynana* Is Controlled by Temperature Experienced during the Pupal and Adult Stages

**DOI:** 10.1371/journal.pone.0064061

**Published:** 2013-05-22

**Authors:** Ashley Bear, Antónia Monteiro

**Affiliations:** Department of Ecology and Evolutionary Biology, Yale University, New Haven, Connecticut, United States of America; Ecole Normale Supérieure de Lyon, France

## Abstract

Environmental cues can act to initiate alternative developmental trajectories that result in different adult phenotypes, including behavioral phenotypes. The developmental period when an organism is sensitive to the cue is often described as a critical period. Here we investigated the critical period for temperature-sensitive courtship rate plasticity in the butterfly *Bicyclus anynana.* We performed a series of temperature-shift experiments in which larvae, pupae, or adults were shifted for blocks of time from one temperature to an alternative temperature, and then we quantified the courtship rate exhibited by adult males. We discovered that the critical period begins during pupal development and extends into adulthood, but temperature experienced during larval development does not affect male courtship rate. This finding allows us to develop hypotheses that address how developmental and physiological factors may have influenced the evolution of behavioral plasticity in this species.

## Introduction

In adaptive phenotypic plasticity, a single genotype produces alternative phenotypes in response to different environments, and each of these phenotypes has a higher fitness in its respective environment than in the alternative environments [1]–[4]. Many adaptive plastic responses are initiated by external environmental cues that the organism receives while it is still in the process of developing to maturity. These cues are usually predictive of the future selective environment and are used to initiate alternative developmental trajectories that result in different adult phenotypes [5]. Understanding how organisms connect external environments with the development of particular traits is, thus, critical for a complete appreciation of how plastic adaptive traits evolve.

Often, there is a specific time interval during development when the environmental cue affects the plastic trait’s phenotype. This period is commonly referred to as the “critical period” [5]. The identification of this critical period of sensitivity to environmental cues is the first step in identifying the initial physiological and/or developmental changes that will regulate trait plasticity. While critical periods have been identified for a number of plastic traits in a variety of species [6]–[10], for many species, it is currently unclear whether the same or different critical periods are used to regulate multiple plastic traits in the same species. By focusing our research on a species of African butterfly, *Bicyclus anynana,* that exhibits plasticity in at least two types of traits: morphological plasticity in wing patterns [10], previously shown to be adaptive [11], and behavioral plasticity in courtship rate [12], we aim to address whether these butterflies have evolved to rely on the same or different critical period for the development of the plastic morphological and behavioral phenotypes.

Laboratory studies revealed that both the morphological and the behavioral plasticity can be induced by differences in rearing temperature that mimic the seasonal fluctuations that these butterflies experience during the wet season (WS) and the dry season (DS) in Malawi [10], [12]. The critical period for the wing pattern plasticity has been previously identified and assigned primarily to the final larval instar [13]. Here we investigate the critical period for the courtship rate plasticity in *B. anynana* and establish that the critical period for this plasticity in behavior is distinct from the critical period for the morphological plasticity.

The *B. anynana* behavioral plasticity examined here involves a change in the rate of the stereotypical courtship behavior of the species during a prescribed observation period (described in [14]). Specifically, a previous study found that when the butterflies were reared at a high temperature (27°C), that mimics the average daily temperatures of the WS, adult males courted females more often during the observation period. However, if the butterflies were reared at a lower temperature (17°C), that mimics the average temperature of the DS, adult males courted significantly less often [12]. Data from a previous study provides evidence that this courtship rate plasticity is associated with changes in the costs and benefits to courtship that are likely mediated by plasticity in male spermatophore quality [12]. Temperature-mediated changes in spermatophore quality, in turn, are correlated with differences in adult resource availability typical of each season in Malawi [10], [12]. The co-variation between seasonal resource availability and courtship rate suggests that adult courtship rate plasticity may be an evolved adaptation to the seasonal environments.

To better understand the proximate, physiological and developmental mechanisms that lead to changes in this adult courtship rate plasticity, we begin here by determining the critical period of temperature sensitivity that leads to variation in the male *B. anynana* courtship rate. We performed a series of temperature-shift experiments in which larvae, pupae, or adults were shifted for blocks of time from the WS or DS temperature (27°C or 17°C, respectively) into the alternative DS or WS temperature, and then quantified the rate of adult male courtship. The results of this study indicate that the temperature *B. anynana* experiences during the pupal period of development, and during adulthood, has a significant effect on the male courtship rate, while the temperature experienced during larval development does not. These results point to the pupal period as an important stage in development for investigating proximate mechanisms of adult courtship plasticity and raise questions about the factors that influence why the critical period begins during this developmental stage.

## Materials and Methods

### Animal Rearing Conditions

The *B. anynana* lab colony stock was originally derived from 80 gravid females collected in Malawi in 1988, and has been maintained at Leiden and at Yale University since then at a size of about 200–300 breeding individuals each generation. *B. anynana* larvae were fed young corn plants and adults were fed mashed banana. WS and DS forms were produced by rearing animals in climate-controlled chambers that differed only in temperature. One chamber was maintained at 27°C and the other at 17°C. Humidity was kept constant at 80% and light: dark cycles were 12 h:12 h in both chambers. Adult butterflies and eggs were routinely moved between the climate-controlled chambers in order to control for possible maternal effects on plastic phenotypes and to ensure that the whole *B. anynana* colony was one inter-breeding population.

### Temperature Shift Experiments

**Figure 1:**
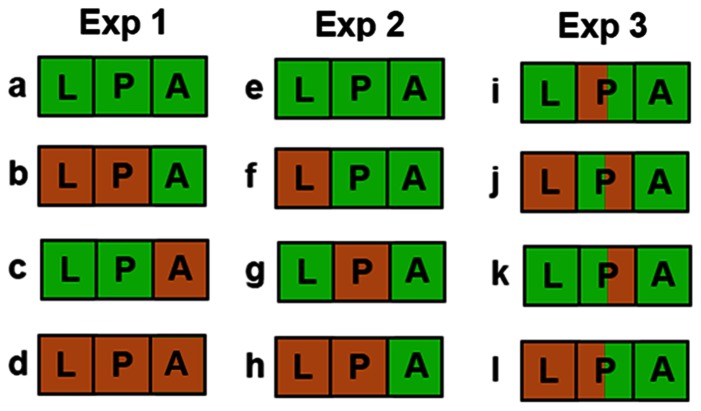
Schematic of the different temperature shift experiments performed in this study. Each temperature shift treatment has been assigned a different letter, and experiments are grouped by specific question and analysis (Exp 1–3). L, P, and A stand for larval, pupal, and adult development, respectively. Brown (the color of dead leaf litter) indicates the DS temperature of 17°C and green (the color of lush vegetation) represents the WS temperature of 27°C.

We performed a series of temperature-shift experiments in order to determine the critical period during development, or adulthood, when external temperature alters male courtship rate (Fig. 1). In the first experiment, the goal was to establish whether temperature experienced during development or during adulthood was the major determinant of male courtship rate. In the second experiment, the goal was to establish whether the temperature experienced during the larval or pupal stage was the major determinant of the male courtship rate. In the third experiment, the goal was to identify whether the temperature experienced during the first or second half of the pupal stage was the major determinant of the male courtship rate. The particular mid-pupation shift used in the third experiment was chosen because during the first half of pupal development there are known differences in the profile of ecdysteroid hormones between WS and DS *B. anynana* [17]. Since these hormones have a known role in regulating reproductive behavior in flies [15], [16]), they could also be important mediators of plastic reproductive behavior in butterflies.

The temperature shifts were performed at consistent times during larval, pupal, and adult periods. Developmental time varied with rearing temperature but we used the day of particular molts to switch animals across temperatures. The only exception is the pupal shift experiment, in which we used percent of development (calculated by the number of days of pupation, divided by the total length of pupation), to shift animals at equivalent developmental stages. The timing of the transitions are detailed below.

#### Experiment 1

At 10 a.m. on the day of adult eclosion, butterflies were separated by sex to ensure that all animals used in the study were virgins. The butterflies were placed in mesh hanging cages with banana and either remained in the same chamber where they were reared, or were moved to the chamber with the alternative temperature (Fig. 1, Exp 1). In this experiment, the courtship rate observations were conducted at both 27°C and 17°C.

#### Experiment 2

At 2 p.m. on the day that animals became pre-pupa, they either remained at the same temperature as that experienced during larval development or were moved to the chamber with the alternative temperature. At 10 a.m. on the day of adult eclosion, butterflies were separated by sex and placed in mesh cages with banana in the 27°C chamber (Fig. 1, Exp. 2). All courtship rate observations were performed at 27°C.

#### Experiment 3

At 50% of pupal development (which was on day 10 after pupation for DS and on day 3 after pupation for WS individuals; day 1 of pupation is the first day when pupae are first visible inside the rearing cages by 2pm - these pupa are ≤20 hours old since they begin to pupate by about 6 p.m. the previous evening), at 2 pm, pupa either remained at the same temperature or were moved to the chamber with the alternative temperature. At 10 a.m. on the day of adult eclosion, butterflies were separated by sex and placed in mesh cages with banana in the 27°C chamber. These pupal shifts were performed with both possible permutations of larval temperature (Fig. 1, Exp. 3). All courtship rate observations were performed at 27°C.

### Estimating Mortality at Different Temperatures

The DS environment is thought to be a more stressful environment than the WS, which could explain the reduced courtship rate of DS reared individuals [18]. Though data from other studies suggest that this is not the case ([12], Westerman et al. unpublished data), in this study, we aimed to further address whether stress could be causing lower courtship rates in DS males by using mortality as a proxy for stress. Thus, we collected a known number of pre-pupae from each rearing temperature and then measured the survivorship of the pupa by counting the number of individuals that eclosed at each temperature.

### Behavioral Observations

On the day of eclosion (day 1), the animals were separated by sex to ensure that all animals used in the study were virgins. All behavioral observations were performed on day 7, when males were placed together with females in net cylindrical hanging cages (30 cm diameter, 40 cm height) at 5 p.m. This time of observation was chosen because *B. anynana* exhibits crepuscular courtship. In the first experiment, courtship observations were performed at both 17°C and 27°C. In all other experiments, however, the courtship observations were made only at 27°C. During the experimental observation period the courtship rate (the number of times males courted during the observation period) was recorded.

In order to address any possible effects of time of year or cohort on male courtship rate, we performed an analysis of co-variance using experimental treatment as a factor and the date of the experiment as a cofactor, and asked whether date explained a significant proportion of the variance in the courtship data. We accounted for unequal variances in courtship across treatments, determined using Levine’s test, by square root transforming the data.

The courtship data for the temperature shift experiments was analyzed using a full factorial two-way analysis of variance (GLM, general linear model) on square root transformed data for each of the experiments separately. Some details of the experimental design, and factors used in the analyses, varied between the shift experiments. These are detailed below.

#### Experiment 1

Ten males and ten females were placed together in a hanging cage. The butterflies were observed continuously for 50 minutes and every courtship event was recorded in order to establish the courtship rate for each treatment. Factors used in the GLM were 1) temperature experienced during larval & pupal development and 2) temperature experienced during the first seven days of adulthood.

#### Experiments 2 & 3

We changed five aspects of the experimental design described above in order to streamline the data collection process. First, we reduced the number of butterflies in each experiment to five males and five females. Second, we placed a UV light source above the observation cage since a previous study found that UV reflective ornaments on *B. anynana* play a role in mate attraction [19]. Third, observations were made in a 30-minute period, rather than a 50-minute period. Fourth, since the results of Experiment 1 indicated that the temperature experienced by the adult butterflies affects male courtship rate, we kept adult temperature constant at 27°C in all other experiments, and the courtship observations were done at this temperature only. Finally, all females used in these experiments were reared at a common temperature (27°C) to control for any possible influence of female phenotype on male behavior. Factors used in the Experiment 2 GLM were 1) temperature experienced during larval development and 2) temperature experienced during pupal development. We combined data from Experiment 2 and Experiment 3 to run a full factorial GLM with three factors: 1) temperature experienced during the first half of pupal development, 2) temperature experienced during the second half of pupal development, and 3) temperature experienced during larval development. All analyses were performed in SPSS Statistics, version 19 for the Mac. Graphs were made in Excel.

## Results

We found that the date on which a particular experiment was performed did not significantly explain variation in male courtship rates (F_1,103_ = 2.384, p = 0.126), whereas experimental treatment did (F_1,103_ = 6.291, p<0.000). Below we examine which stage of development or of adulthood was the critical period of temperature sensitivity for explaining male courtship rate variation.

### Both Developmental and Adult Temperatures Influence Adult Male Courtship Rate

**Figure 2:**
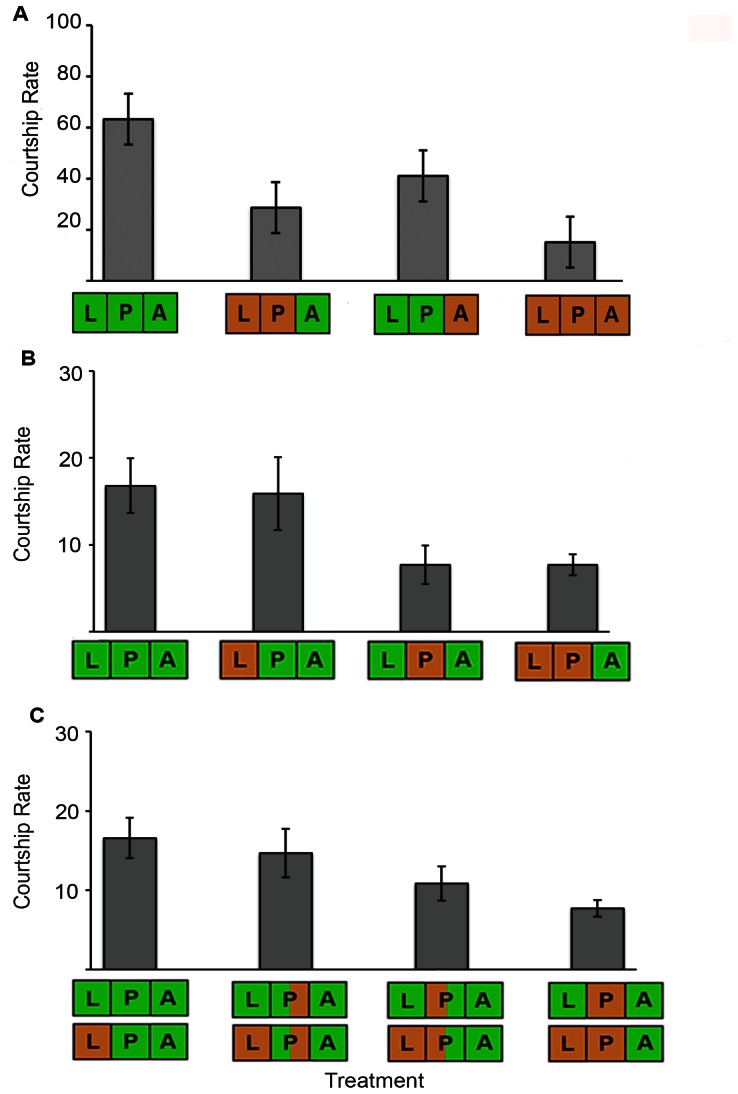
Courtship rate is influenced by the temperature the butterfly experiences during pupation and adulthood, but not during larval development. Vertical bars represent the mean courtship rate exhibited by males per observation period. Error bars represent 95% confidence intervals. The temperature treatment the butterfly experienced during development and adulthood is represented by horizontal bars along the x-axis. Symbols used below the x-axis are defined in Figure 1. A: Bars represent the mean courtship rate of ten males in a 50-minute observation period. B, C: Bars represent the mean courtship rate of five males in a 30-minute observation period. C: Presents the mean courtship rate from pooled data from both types of larval temperature experienced during development (larval temperature was not a significant factor in the GLM analysis).

The data collected for Experiment 1 showed that the temperature a butterfly experiences during its development significantly affects courtship rate (F_1,38_ = 41.92, p<0.000), as does the temperature a butterfly experiences during adulthood (F _1,38_ = 15.14, p<0.000). Males that spent adulthood at 27°C had a higher courtship rate than males that spent adulthood at 17°C. In addition, males that developed at 27°C exhibited a higher courtship rate than males that developed at 17°C (Fig. 2A).

### Temperature Experienced during the Pupal Stage, but not the Larval Stage, Influences Adult Courtship Rate

Data collected for Experiment 2 showed that the temperature experienced during the pupal stage had a significant effect on courtship rate (F_1,66_ = 33.88, p<0.000), while temperature experienced during larval development did not (F_1,66_ = 0.022, p = 0.883). Animals reared at higher temperature during the pupal stage had a higher courtship rate than animals reared at lower temperature (Fig. 2B).

Data collected for Experiments 2 and 3 combined showed that the temperature experienced during both the first (F_1, 104_ = 26.80, p<0.000) and second half (F_1, 104_ = 4.28, p = 0.041) of pupal development had a significant effect on male courtship rate, but temperature experienced during larval development, again, did not (F_1, 104_ = 0.069, p = 0.409). While high temperatures experienced both during the first and second halves of pupation increased male courtship rate, the higher F values and courtship means resulting from temperature exposures during the first half of pupation suggest that this time interval of pupal development is critical for shaping adult male courtship phenotypes (Fig. 2C).

### There is no Difference in Mortality between WS and DS Reared Pupae

Data collected on the mortality of WS and DS reared pupae demonstrates that there is no difference in mortality in these different rearing conditions (In both the 27°C and 17°C chambers, 20 out of 23 individuals, or 87%, eclosed).

## Discussion

The African butterfly *B. anynana* exhibits plasticity in male courtship rate that can be induced by the temperature experienced during development and adulthood. A previous study suggested that plasticity in courtship rate in *B. anynana* males evolved due to variation in the costs to mating in the WS and DS conditions [12]. Mating costs are thought to be higher in the DS because DS season males give females a higher quality spermatophore and have, as a consequence, shorter lives [12]. In this study, we manipulated when butterflies were exposed to representative average WS and DS temperatures in order to identify the critical period when temperature modifies adult male courtship rate. We found that the temperature experienced during pupal development and adulthood, but not larval development, modifies adult courtship rate.

One possible interpretation for the influence of the temperature experienced during adulthood on male courtship rate is that the critical period for the *B. anynana* behavioral plasticity extends throughout development into adulthood. An alternative interpretation is that the temperature experienced during adulthood could be having a direct effect on locomotion because insects are ectothermic. This latter interpretation would explain why we find the same relative differences in courtship rate when we compare the behavior of individuals that experienced the same adult conditions but different developmental conditions (see Fig. 2A). Nevertheless, in either possible scenario, the results of this study indicate that the alternative developmental trajectories that modify adult male courtship rate begin during the pupal stage under the influence of environmental rearing temperature. These results raise questions about how and why the critical period begins during this stage of development.

Theoretically, organisms that can adaptively match their phenotype to the environment where they will live should have a fitness advantage relative to non-plastic organisms. In the case of organisms that live in predictably fluctuating environments, where different phenotypes are favored in different environments, organisms should evolve plastic development that responds to cues that are good predictors of the future environment [1]. Plastic development makes a lot of sense for morphological traits that are specified and differentiated before adulthood, but for behavioral phenotypes, which tend to be highly labile, we might expect that the best indicators of the future environment are the cues present in the future environment itself. This raises questions about how and why an organism’s adult behavioral phenotype is modified in response to a predictive cue at an earlier time in development.

How is temperature during development influencing the adult courtship rate in *B. anynana?* Could it be due to a stress response to temperature? Is it due to an alteration in brain development during pupation? Might endocrine glands develop differently at different temperatures leading to different effects of hormones on the brain during development, adulthood, or both? Though we do not have conclusive answers to these questions at this time, we have data that allows us to speculate about which mechanisms might be at play.

Recent data on *B. anynana* courtship rate, locomotion, and mortality rate, suggest that the cooler DS temperatures experienced in the lab in this and other studies do not produce a stress response that decreases adult courtship rate. For example, Prudic et al. (2011) found a sex-role reversal in which the courtship rate of females reared at the lower DS temperature was significantly higher than that of females reared at the WS temperature, while the opposite was the case in males (as in the present study). These results imply that the lower rearing temperature does not lead to lower courtship rates due to stress, at least in the case of females. In addition, a recent study by Westerman et al. (in review) showed that locomotion (which is broken down into four different quantifiable behaviors) is actually higher in DS males and females than WS males and females when single pairs of butterflies are observed at a common temperature a few days after eclosion (Westerman et al, in review). Finally, the fact that in the present study we found no difference in mortality between WS and DS reared butterflies, suggests that the DS temperature may not, on its own, be stressful to the butterflies (see Results section). Taken together, the data provided by Prudic et al. 2011, Westerman et al. (in review), and from the present study, suggest that the low DS temperatures do not induce lower courtship rates due to stress.

Another possible mechanism by which temperature during development could lead to changes in adult courtship rate is through altered brain development and/or altered development of traits that influence brain development and/or adult behavior, such as endocrine glands. Support for this hypothesis comes from the discovery that the critical period for adult courtship rate plasticity begins during the pupal period, which incidentally, is also a time in insect development when the brain and endocrine glands undergo major metamorphosis [20], [21]. This correlation is particularly interesting when considered in light of previously published data on the critical period for temperature-cued plasticity of a morphological trait (ventral eyespot size). While, the present study showed that the temperature-sensitive critical period for the courtship rate plasticity begins during pupal development, a previous study showed that the temperature-sensitive critical period for ventral eyespot size plasticity is primarily during the late larval instar and first hours of pupation [13]. The difference in the timing of the critical periods for cuing plastic development in each of these traits is interesting and may highlight critical windows in eyespot and behavioral development that are particularly amenable to alteration by an environmental cue, or in other words, critical windows in trait development that, if altered, can result in profound phenotypic change. For instance, during late larval development eyespot centers are being differentiated [22]–[26], and by 24 hrs after pupation, signals from the center appear to have been read by transcription factors that define the size of the future eyespot rings [27]–[31]. Gene expression differences scored between DS and WS butterflies showed that this period in development (from late larvae to early pupae) establishes changes in the extent of expression of at least one transcription factor, Distal-less, in the eyespot field [26]. These data suggest that the time-course of eyespot size differentiation, as monitored by plastic changes in gene expression patterns, happens in concert with the critical period of wing pattern environmental sensitivity. Little is currently known about developmental factors that influence behavior in *B. anynana,* but our own observations, as well as experiments in other insects, show that the brain and endocrine glands undergo a major metamorphosis during the pupal stage [20]–[22]. This suggests that, just as ventral eyespot size regulation responds to a cue at the time when the ventral eyespots are developing, *B. anynana* behavioral regulation is responding to an environmental cue at the time when the brain and endocrine glands are being developmentally remodeled. This correlation between the critical period for the courtship plasticity and the restructuring of the brain and endocrine glands during pupation points to a possible role for plastic endocrine and/or brain development in producing the observed changes in courtship rate between the two seasonal forms.

Regardless of which factors directly influence plasticity in courtship rate in WS and DS *B. anynana* males, the environmental sensitivity during the critical period is likely to affect courtship rate indirectly via hormones, as seen in a variety of different insect systems [5]. A recent study in *B. anynana* found that in both DS and WS development there is an ecdysteroid hormone titer peak during the first half of pupation, but the titers peak earlier in the WS versus the DS forms, even when controlling for different developmental rates across seasonal forms [17]. This hormone peak shift in *B. anynana* was initially examined in connection to plasticity of ventral eyespot size, however, the authors concluded that changes in the timing of the hormone titer peaks alone could not regulate the development of alternative wing patterns. They suggested, rather, that these hormone titer shifts are either not involved in regulating wing pattern plasticity or that there is a complex interplay between the hormone titer shifts and the hormone receptor expression that regulates the development of the wing pattern phenotype. We propose that an alternative possibility is that the endocrine shifts described in that study may instead be regulating the courtship rate plasticity. Ecdysteroids are a class of hormones that coordinate the metamorphosis of the larval brain into the adult brain in insects [20], and also regulate courtship behavior in *Drosophila melanogaster* [15], [16]. We are currently manipulating ecdysteroid titers during pupal development to test whether these hormones are sufficient to alter the courtship rate of the adult butterflies, irrespective of rearing temperature.

Another important question to address is *why* temperature experienced during pupal development has such a large effect on the courtship rate in *B. anynana* males. One possible answer is that developmental plasticity may increase the efficiency of adult phenotype-environmental matching for at least two reasons. First, if there is a time lag between the temperature experienced by the adult butterfly and the modification of the courtship phenotype, this could lead to temporary poor phenotype-environment matching. By responding to temperature during pupation, the butterflies may be primed to exhibit the appropriate courtship phenotype upon eclosion. Second, developmental plasticity during pupation could modify brain development or the development of structures that communicate with the brain, such as endocrine glands, in a way that enhances or even elicits the adult courtship phenotype [20]–[22]. The retention of adult sensitivity to temperature, on the other hand, allows butterflies that develop during transitional periods between the WS and DS (when the temperature during pupation may not sufficiently predict the environment the butterfly will experience during adulthood) to still produce the appropriate courtship phenotype.

### Conclusion

The results presented here demonstrate that the critical period for male courtship rate plasticity in *B. anynana* begins in the pupal stage and extends into the adult stage. This is in contrast to the critical period for the *B. anynana* morphological plasticity, which occurs primarily during late larval development. *B. anynana* has, thus, evolved different critical periods of temperature sensitivity for different plastic traits. The specific timing of the critical period for plastic courtship rate in *B. anynana* raises questions about the proximate (how) and ultimate (why) factors that dictate when a critical period evolves. Establishing the critical period of environmental sensitivity via shift experiments, as done here, may also directly highlight the relevant stages in trait development that are responsive to the environmental cues. This information will enable us to focus future physiological and genetic studies, such as investigating the role of hormones in regulating behavioral plasticity and their primary target genes, on that specific developmental window.
